# Preimplantation genetic diagnosis and screening (PGD/S) using a semiconductor sequencing platform

**DOI:** 10.1186/s40246-018-0187-x

**Published:** 2019-01-03

**Authors:** Li-Ya Wang, Xing-Qiang Rao, Yu-Qin Luo, Bei Liu, Chun-Fang Peng, Dan Chen, Kai Yan, Ye-Qing Qian, Yan-Mei Yang, Ying-Zhi Huang, Min Chen, Yi-Xi Sun, Hong-Ge Li, Ying-Hui Ye, Fan Jin, Hai-Liang Liu, Min-Yue Dong

**Affiliations:** 1 Key Laboratory of Reproductive Genetics, Ministry of Education (Zhejiang University) Women’s Hospital, School of Medicine, Zhejiang University, Hangzhou, 310006 China; 2 CapitalBio Genomics Co., Ltd., Dongguan, 532808 China

**Keywords:** Preimplantation genetic diagnosis/screening, Semiconductor sequencing platform, Array comparative genomic hybridization, Whole genome amplification, Copy number variation

## Abstract

**Background:**

Recent advances in semiconductor sequencing platform (SSP) have provided new methods for preimplantation genetic diagnosis/screening (PGD/S). The present study aimed to evaluate the applicability and efficiency of SSP in PGD/S.

**Methods:**

The artificial positive single-cell-like DNAs and normal single-cell samples were chosen to test our semiconductor sequencing platform for preimplantation genetic diagnosis/screening (SSP-PGD/S) method with two widely used whole-genome amplification (WGA) kits. A total of 557 single blastomeres were collected from in vitro fertilization (IVF) couples, and their WGA products were processed and analyzed by our SSP-PGD/S method in comparison with array comparative genomic hybridization (array-CGH).

**Results:**

Our SSP-PGD/S method indicated high compatibilities with two commercial WGA kits. For 557 single blastomeres, our method with four million reads in average could detect 24-chromosome aneuploidies as well as microdeletion/microduplication of the size over 4 Mb, providing 100% consistent conclusion with array-CGH method in the classification of whether it was transplantable.

**Conclusions:**

Our studies suggested that SSP-PGD/S represents a valuable alternative to array-CGH and brought PGD/S into a new era of more rapid, accurate, and economic.

**Electronic supplementary material:**

The online version of this article (10.1186/s40246-018-0187-x) contains supplementary material, which is available to authorized users.

## Introduction

In vitro fertilization (IVF) is the basis for successfully selecting a viable embryo after ovarian stimulation [1]. Studies have shown that approximately 25% of oocytes in women in their early 30s are chromosomally abnormal [2]. Moreover, the proportion of aneuploidy of the oocytes increased to more than 75% among women over 40 years old [3]. Embryonic chromosomal abnormalities can directly lead to implantation of an abnormal conceptus, resulting in early miscarriage, late abortion, or the delivery of an affected child with a trisomy or monosomy [4]. It could significantly decrease the proportion of successful pregnancies in women over the age of 35 [5, 6].

Compared with couples with normal karyotype, individuals who were diagnosed as translocation carriers had a higher ratio of inherited copy number variations (CNVs) in their embryos, such as the abnormal of chromosome 13, 14 in the blastomeres of robertsonian translocation carrier [7]. Robertsonian translocation carriers were phenotypically normal but had a high risk of miscarriage and may have a child with chromosomal abnormalities [8, 9]. Accurate diagnosis can increase the success rate of implantation and live birth and reduce the miscarriage.

Preimplantation genetic screening/diagnosis (PGS/D) is used to identify genetic defects of early embryo [10–12]. The first standard PGD/S technique for chromosomes detection was fluorescence in situ hybridization (FISH). The specific probes for aneuploidy screening are mainly aimed at the common problems in fetus [13]. FISH can only test a minority of chromosomes, which may omit other chromosomal aneuploidy. Nowadays, it has widely been replaced by microarray techniques that target all chromosomes [14]. Array comparative genomic hybridization (array-CGH) in PGS screening has been proven to be a golden standard for aneuploidy detection [15]. However, PGD/S solution is constantly being updated with the advent of next-generation sequencing (NGS) [16, 17].Whole-genome amplification (WGA) offers means to enrich DNA quantities before sequencing library preparation due to the limitation of the picogram amounts of DNA in single cells [18]. The two most widely used commercial WGA kits were chosen for the evaluation of single-cell WGA-NGS approaches in this study.

Semiconductor sequencing is one of the next-generation sequencing methods. It has some advantage such as rapid, long reads and convenient, comparing to illumina platform. The present study aimed to evaluate the applicability and efficiency of SSP for PGD/S. The study was divided into two parts. In the first part, we diluted artificial single-cell-like DNA samples from abortive villi to test the performance of our semiconductor sequencing platform for preimplantation genetic diagnosis/screening (SSP-PGD/S) and its compatibility with two popular WGA kits. Variation detection was performed, and coefficients of variance (CV) in 15-pg DNA samples were calculated to evaluate the two popular WGA kits with our SSP-PGD/S method. In the second part, 557 single blastomeres were collected from 157 couples, mostly with abnormal karyotype existing in either of them. All of the WGA products were processed and analyzed by both our SSP-PGD/S method and array-CGH to evaluate the accuracy and reliability of our method.

## Materials and methods

### Subjects

This study was organized into two parts with regard to the experiments. The first part involved an evaluation of karyotypically defined chromosomally abnormal single-cell DNA deriving from tissues with chromosomal abnormalities. The second part involved a retrospective blinded assessment of WGA products, selected from 157 consecutive clinical PGS cycles performed on single blastomeres that were biopsied from cleavage-stage embryos in the period of July 2014–November 2016. The karyotype of parents was shown in Additional file 1: Table S4, and therefore, we can analyze whether the CNV in embryo is inherited or de novo. Written informed consent was obtained from all participants in this study. This study was approved by the Ethics Committee of Women’s Hospital, School of Medicine, Zhejiang University.

### Artificial single-cell-like DNA sample preparation

We extracted DNA from abortive villi samples from 21 cases with chromosomal abnormalities. The types of chromosomal abnormalities are listed in Additional file 1: Table S1. And a sample of lymphocytes of a normal male is also used. The DNA concentration was determined by the Qubit dsDNA High Sensitivity Assay kit (Life Technologies, Carlsbad, USA). Then, DNAs were diluted to 15 pg/μl. Next, 1 μl DNA was prepared for whole genome amplification using two commercial kits based on DOP-PCR or SurePlex strategies. The chimeric samples were mixed using two DNAs of different karyotypes. Three nanograms of one DNA sample and 6 ng of the other were mixed and then diluted to 60 pg/μl, with 1 μl used for whole genome amplification. The kits used in this study were the Genome Plex® Single Cell WGA Kit (DOP-PCR) and the Rubicon Genomics Pico PLEX® WGA Kit (SurePlex). All of the experimental operations followed the manufacturer’s instructions.

### WGA of single-cell genomic DNA with different WGA kits

Single blastomere was isolated as described previously. Briefly, following sufficient dissociation and dilution of cells, single cell was randomly picked up using a mouth pipette under a microscope and washed three times in phosphate-buffered saline to avoid exogenous DNA contamination, after which they were transferred into a PCR tube. Single-cell isolation was confirmed by microscopy to ensure that only one cell was inside each tube. After the sample collection, biopsied blastomere was subjected to WGA amplification for both SSP-PGD/S and array-CGH analysis using SurePlex kits (BlueGnome).

### SurePlex WGA

WGA products were processed according to the BlueGnome 24sure plus protocol. Cell lysis and amplification was performed following the manufacturer’s instructions using the SurePlex Amplification system (Bluegnome, Cambridge, UK). As a positive control, 2.5 μl of female control DNA (G1521; Promega; 187 ng/μl) was used at a concentration of 25 pg/μl. The blank was equal to 2.5 μl of PBS. All of the samples were purified using Agencourt® AMPure® XP (Beckman Coulter, cat. no. A63881) according to the manufacturer’s protocol. The concentration was measured using the Qubit dsDNA High Sensitivity Assay kit (Life technologies, Carlsbad, USA).

### Array-CGH experiment and analysis

All of the samples, amplified by SurePlex, were analyzed using the 24-Sure+ array (Bluegnome). The CNAs observed on this array should also be observed on the reference array profile. The array-CGH was performed according to the 24-Sure+ Protocol (Bluegnome), with a male genomic DNA sample as the reference. Copy number calls automatically generated by the Bluefuse Multi (Bluegnome) were assessed manually. The BlueFuse algorithm was based on calculating the median log2 ratio of all of the chromosomes for the detection of gains and losses. A median log2 ratio value of 0.3 or more indicated chromosome gains, whereas values of − 0.3 or less indicated chromosome losses.

### Dop-pcrwga

Cell lysis and amplification were performed using the GenomePlex Single Cell WGA Kit (Sigma-Aldrich WGA4, Darmstadt, Germany) following the manufacturer’s instructions. As a positive control, 1 μl of male control DNA was used with a concentration of 15 pg/μl. The DNA samples used in this step were the same as the positive control. The blank was 1 μl of PBS. All of the samples were purified and measured following the manufacturer’s protocol, as described previously.

### SSP-PGD/S analysis

WGA amplification products were ligated to sequencing adapters using kits in accordance with the manufacturers’ protocols. In brief, 200 ng of the WGA products was fragmented to an average size distribution of 150 bp with the S2 Focused Ultrasonicator with Adaptive Focused Acoustics (AFA) technology (Covaris, Woburn, USA). Subsequently, libraries of the fragmented samples were created using the Ion Xpress™ Plus Fragment Library Kit, following the manufacturer’s protocol. Sequencing was performed on Ion Proton (Thermo Fisher Scientific, MA, USA). A total of 15 samples with each a different index were multiplexed on one chip. Samples were pooled at 20 nM each and diluted to a final concentration of 20 pM. Sequencing of 15 samples at 150 bp on an Ion Proton should lead to average genome coverage of 0.25× per sample (5 M reads/sample).

Reads were aligned to the human genomic reference sequences (hg19) using the BWA. Reads that were unmapped or had multiple primary alignment records were filtered in the alignment file, using an in-house Perl script. Duplicate reads were also removed. To eliminate the effect of WGA and sequencing bias, a two-step correction process was applied. In the first step, LOESS regression of the reads ratio of each 20 kbbin was used to smooth the GC bias. Then, in the second step, bins of outliers were masked to remove bias from the WGA. Further, chromosomal aneuploidies were detected by the *Z*-score method in comparison with control samples. *Z*-score > 3 was considered to be trisomy, while *Z*-score < − 3 was considered to be monosomy. Candidate CNVs (copy number variations) were detected by the CBS algorithm based on ratios of each 500 kbbin, which were accumulated by corrected values of 20 kbbins. The segments that had a reads ratio of< 1.4 were considered to be microdeletions, while the segments that had a reads ratio of > 2.6 were considered to be microduplications. The CNVs were selected by *P* value < 0.01 for a 10,000 times random permutation. Embryos were considered to be “implantable” if there were no aneuploidies or CNVs.

## Results

### Performance of SSP-PGD/S method on artificial samples

The artificial positive single-cell-like DNA (detail in materials and methods, also called 15-pg DNA) and normal single-cell samples were amplified using the SurePlex and DOP-PCR kits, to test the performance of our SSP platform and its compatibilities with WGA kits. The types of artificial samples are listed in Additional file 1: Table S1. To evaluate stability for SSP, the GC content (GC%), the CV for bins with sizes of 1 Mb and the ratio of duplication reads were calculated (Table 1). We found no significant difference in the GC content between the two kits (45% for SurePlex and 41% for DOP-PCR). The duplication ratio of the 15-pg DNA samples was no significant difference (19% for SurePlex and 20% for DOP-PCR, *p* > 0.05). The CVs were both low in the 15-pg DNA samples, which indicated high consistency. Interestingly, similar results were also found in artificial single-cell samples. CVs of SSP-PGD/S method had similar result with Xie’s group [19]. There were no failed samples (reported with > 5 aneuploidies), and no significant differences were found on the detectable aneuploidy, CNVs, mixing aneuploidy and mixing CNVs between the two kits, as shown in Table 2. Moreover, none of false positive events were identified in both two kits.

**Table 1:** Deep-sequencing statistics for the 15-pg DNA and single-cell samples amplified by different kits

Base index	Sample types	Kits (mean ± SD)		Kits (no. of samples)	
		SurePlex	DOP-PCR	SurePlex	DOP-PCR
GC content		0.448 ± 0.004	0.414 ± 0.006	26	66
CV(1 Mb)	15 pg DNA	0.133 ± 0.01	0.11 ± 0.014	12	25
	Single-cell DNA	0.117 ± 0.012	0.094 ± 0.013	3	4
Duplication ratio	15 pg DNA	0.187 ± 0.039	0.199 ± 0.059	12	25
	Single-cell DNA	0.185 ± 0.052	0.185 ± 0.05	3	4
Unique mapped reads ratio	15 pg DNA	0.517 ± 0.01	0.515 ± 0.057	12	25
	Single-cell DNA	0.523 ± 0.061	0.479 ± 0.023	3	4
Unique mapped reads number	15 pg DNA	4,137,564 ± 517,233	3,953,195 ± 650,395	12	25
	Single-cell DNA	2,883,315 ± 241,218	3,574,140 ± 797,115	3	4
Theory coverage	15 pg DNA	0.215 ± 0.025	0.194 ± 0.034	12	25
	Single-cell DNA	0.162 ± 0.015	0.179 ± 0.036	3	4
Actual coverage	15 pg DNA	0.133 ± 0.012	0.102 ± 0.018	12	25
	Single-cell DNA	0.108 ± 0.008	0.096 ± 0.017	3	4
Minus of theory and actual	15 pg DNA	0.082 ± 0.02	0.091 ± 0.033	12	25
	Single-cell DNA	0.054 ± 0.008	0.083 ± 0.025	3	4

**Table 2:** Aneuploidy and CNV detection in 15-pg DNA samples amplified using different WGA kits

CNV type	Mixing proportion	CNV length	Kits	
			SurePlex	DOP-PCR
Aneuploidy	1	NA	2/2	2/2
CNV	1	< 4 Mb	4/4	4/4
		4–10 Mb	1/1	1/1
		> 10 Mb	1/1	1/1
Mixing CNV	0.3	< 4 Mb	0/1	0/1
		4–10 Mb	2/3	2/3
		> 10 Mb	8/9	9/9
	0.7	< 4 Mb	0/0	0/0
		4–10 Mb	5/5	5/5
		> 10 Mb	8/8	8/8
Mixing Aneuploidy	0.3	NA	4/4	4/4
	0.7	NA	4/4	4/4

### Conformance analysis of single blastomere with SSP-PGD/S and array-CGH

A total of 157 patients with a mean age of 30 years who met the inclusion criteria were enrolled in the study. A total of 557 single blastomeres were successfully assessed by both our SSP-PGD/S method and array-CGH methods (Additional file 1: Table S2).

**Fig. 1:**
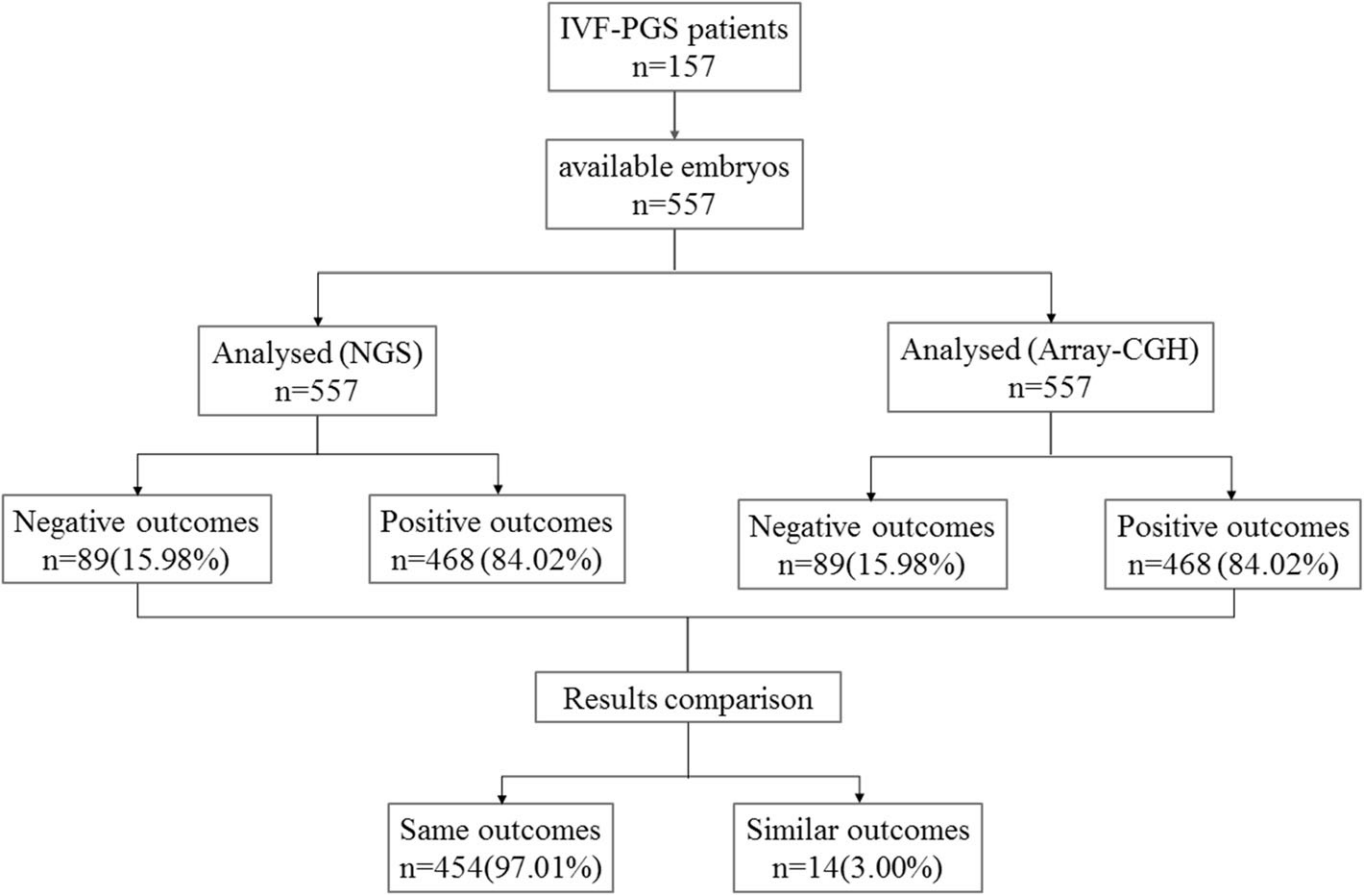
Recruitment and testing algorithms for IVF-PGS patients

The consistency between the SSP-PGD/S and the array-CGH results was calculated with the classifications of positive or negative (Fig. 1). SSP-PGD/S could provide the results of aneuploidies (referring to loss or gain of the whole chromosome) and CNVs (referring to duplications and deletions of the sub-chromosome). Of the samples diagnosed by our SSP-PGD/S method, 16.39% presented negative outcomes with normal diploid, and 83.61% showed concordant outcomes with chromosomal abnormalities. Qualitative analysis of the two groups showed that the results are consistent. Here, we found that 14 positive samples were not identically the same with the typical gains and/or losses for one or more chromosomes between the two platforms (Additional file 1: Table S3). The karyotype of embryos was shown in Additional file 1: Table S5.

### Aneuploidy analysis

**Fig. 2:**
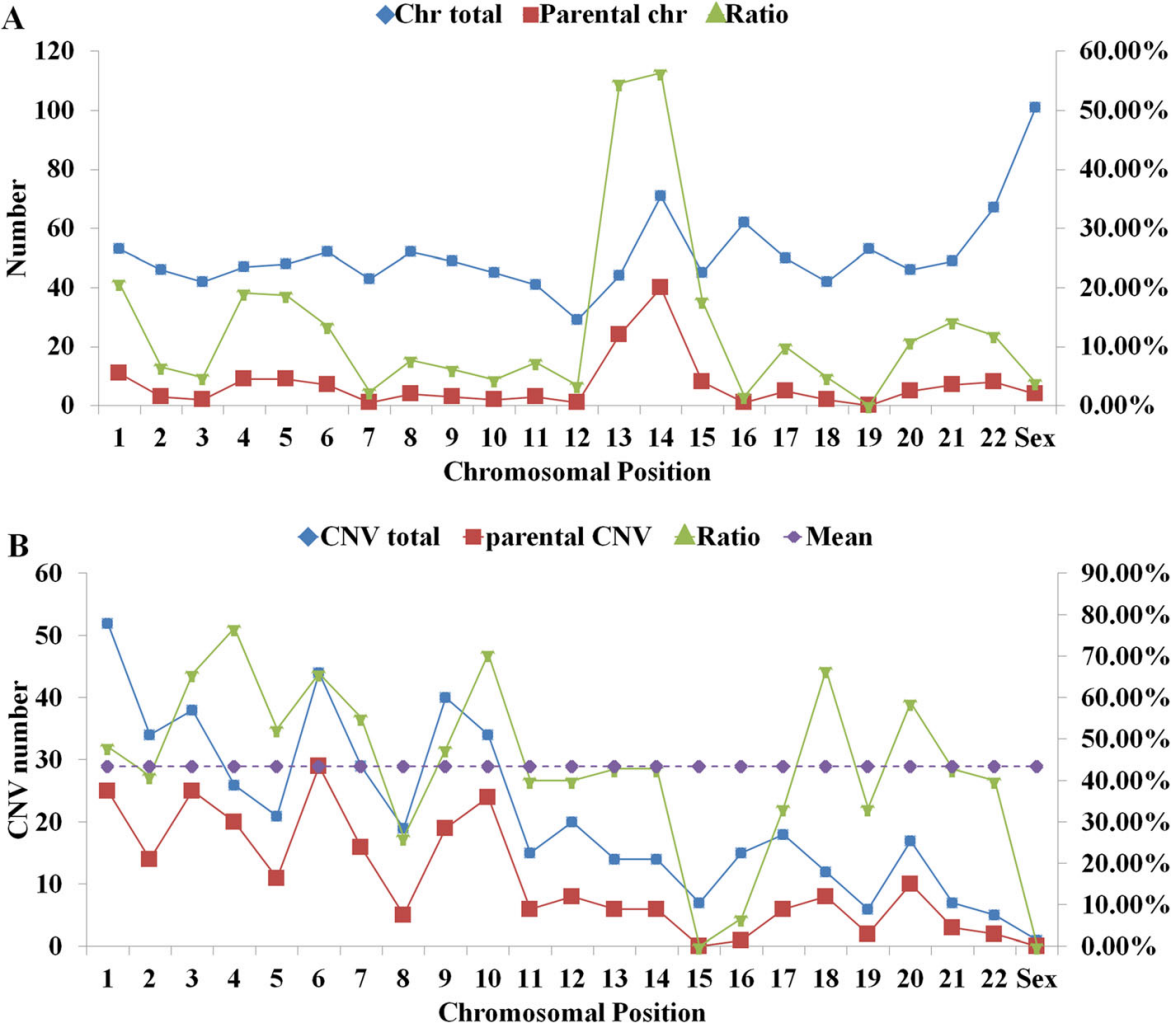
Inherited aneuploidy and CNVs in NGS. **a** Inherited 24-chromosome aneuploidy. **b** 24-chromosome analysis of inherited CNVs performed in the IVF couples with inherited disorder. Blue indicates the number of chromosome aneuploidy/CNVs observed on each chromosome of embryo. Red indicates the number of chromosome aneuploidy/CNVs observed on 2 each chromosome of the parents. Green indicates the inherited probability of chromosome aneuploidy/CNVs

Our results show that each chromosome in the embryo has a risk of aneuploidy. Total 83.61% (454/557) embryos were found to have one or more aneuploidies. We analyzed samples with consistent results between NGS and array CGH. The sex chromosome has the highest probability of non-inherited aneuploidy, with its high incidence and low inherited ratio. Autosomes 14, 16, and 22 also have the high detection rates of aneuploidies. The incidence of inherited aneuploidy in chromosome 13 and 14 is much higher than that of other chromosomes, with the inherited ratio above 50% (Fig. 2a). Autosomes 7, 16, 19 have the high incident rate of non-inherited aneuploidies. Classified by the patient karyotype, the inherited ratio of aneuploidy in translocation and Robertsonian translocation carriers was obviously higher than that of inversion carriers and patients with sex chromosome abnormality (Table 3).

**Table 3:** Correlation of different types of aneuplodies and CNVs between chromosomally unbalanced embryos and patients with abnormal chromosome(s)

Classifications	Aneuplodies						CNVs					
	T	Inherit	Ratio	D	Inherit	Ratio	dup	Inherit	Ratio	del	Inherit	Ratio
t	269	37	13.75%	250	41	16.40%	143	105	73.43%	181	128	70.72%
rob	189	30	15.87%	200	42	21.00%	35	2	5.71%	34	0	–
inv	45	2	4.44%	37	3	8.11%	18	5	27.78%	30	6	20.00%
XY	31	3	9.68%	26	1	3.85%	7	0	–	9	0	–
PGS	68	0	–	62	0	–	15	0	–	16	0	–

Note: *t* Translocation, *Rob* Robertsonian translocations, *inv* inversion, *XY* XY abnormality, *T* trisomy, *D* monosomy, *dup* duplication, *del* deletion, *CNVs* copy number variations

### CNVs analysis

The incidence of inherited CNVs in each chromosome is generally higher than that of aneuploidy, except for 13, 14, 15 and sex chromosomes. The incidence of inherited CNVs is more than 40%. In our study, chromosome 15 and sex chromosome had no inherited CNVs (Fig. 2b). Classified by the length of CNVs, we found that the incidence of inherited CNVs shorter than 100 Mb was about 50%. The incidence of inherited CNVs longer than 100 Mb is significantly increased, due to the high incidence (81.48%) of inherited microdeletions (Table 4). From Table 3, we can see that these inherited CNVs mainly come from embryos of translocation carriers, with 73.43% of the microduplication ratio and 70.72% of the microdeletion ratio. The second is the embryo of the inverted carrier.

**Table 4:** CNVs analysis of data obtained by NGS for the identification of chromosome microdeletions and microduplications

Classes	Micoduplications + microdeletions			Microduplications			Micodeletions		
	Total	Inherit	Ratio	Total	Inherit	Ratio	Total	Inherit	Ratio
1~10 Mb	19	9	47.37%	12	6	50.00%	7	3	42.86%
10~30 Mb	122	58	47.54%	63	27	42.86%	59	31	52.54%
30~50 Mb	132	68	51.52%	50	28	56.00%	82	40	48.78%
50~100 Mb	156	77	49.36%	71	39	54.93%	85	38	44.71%
> 100 Mb	49	34	69.39%	22	12	54.55%	27	22	81.48%

We also showed two representative examples of translocations among the PGD patients here. For a couple with a 46, XX, t (4; 18) balanced translocation, SSP-PGD/S method precisely identified dup18 and del4 segmental imbalances in their single blastomere. In the blastomere from a carrier with 45, XX, rob (13; 14), del 14 was also detected (Additional file 1: Figure S1).

## Discussion

In this study, our SSP-PGD/S method showed a high compatibility and a favorable detection efficiency of two commercial WGA kits with our SSP method, without any failed samples. The low CVs of the SurePlex and DOP-PCR kits indicated their high reliability. Our SSP platform was able to detect a chimerism sample of 30%, whether it was an aneuploidy or CNVs (> 4 Mb). It indicated that diagnosis of CNVs was well within the ability of this technology. Moreover, our method was capable of detecting a case with minimum region of 2.18 Mb bin.

A retrospective blinded assessment of 557 WGA products obtained from single blastomere was performed by SSP-PGD/S method and array-CGH. The comprehensive aneuploidy screening by SSP-PGD/S and array-CGH demonstrated a 100% consistency for the negative and positive estimation, which indicates the accuracy and reliability of SSP-PGD/S method. Furthermore, 14 positive blastomeres did not show the same result between SSP-PGD/S and array-CGH data. This may be related to the quality of the embryo and the concentration of WGA product. The results above clearly demonstrated the ability of SSP-PGD/S methods to provide a direct diagnosis of aneuploidy similar with other NGS methods [20].

The 24-chromosome aneuploidy screening indicated that chromosomes sex chromosome had the highest incidence of aneuploidies. The incidence of inherited aneuploidy of chromosome 13 and 14 is higher than that of other chromosomes. Previous reports showed that NGS was highly sensitive and specific for the detection of 24-chromosome aneuploidy [17, 21, 22]. However, our study suggests that SSP-PGD/S can detect inherited CNVs with a length more than 2.18 Mb as well as 24-chromosome aneuploidies (Additional file 1: Table S1). In the clinical validation study, SSP-PGD/S can accurately and reliably detect the microdeletion/micoduplication of embryos and avoid false positives by comparing the results with their parents’ karyotype.

In our study, we found that, besides with aneuploidies and CNVs inherited from parents’ abnormal karyotypes, listed as robertsonian translocation, translocation, inversion and CNVs, a high incident of de novo aneuploidies and CNVs occurred during meiosis and embryo maturation. For example, autosomes 7, 16, and 19 that have low inherited rate have the high incidence in PGD/S results, while small CNVs have higher incidence in de novo samples than that in inherited samples. Large data of SSP applied in PGD/S could give an alternative route to study chromosomal rearrange mechanism. Weckselblatt et al. [23] reviewed that non-allelic homologous recombination (NAHR) between paralogous long interspersed nuclear element (LINE) or human endogenous retrovirus (HERV) repeats as a cause of deletions, duplications, and translocations.

It must be mentioned that there are several limitations in this study. Although two commercial WGA kits were demonstrated to have high stability and efficiency in our SSP-PGD/S method, and there were no failed samples in our performance testing experiments using artificial single-cell samples and single-cell-like DNA samples, more samples will be required to improve the stability for WGA amplification and sequencing. To better investigate the detection rate of CNVs using SSP method, shorter bins and higher sequencing depth will be needed when testing samples with smaller CNVs.

In addition, another common concern of the PGD result interpretation is chimeras [24, 25]. Routine day 5 biopsies typically contain approximate 5 cells. For that, the lowest possible percent of abnormal cells is 20% (1 of 5 cells) and the highest is 80% (4 of 5 cells). Thus, the ability to distinguish percent of abnormal cells > 80% or < 20% has not been subjected to rigorous validation, and considering that it is not mathematically possible to have any abnormal cells for Controversies in Preconception, Preimplantation, and Prenatal Genetic Diagnosis (COGEN) and the Preimplantation Genetic Diagnosis International Society (PGDIS). Here, we selected a sample of 30% chimeric proportions. It is often overlooked in single blastomere and leads to misdiagnosis. Although some chimeras can be detected by SSP, if there is no chromosomal abnormality in the detected blastomere, it can only be diagnosed by amniocentesis after pregnancy.

The SSP-PGD/S method represents a valuable alternative to array-CGH, with the potential to provide accurate copy number analysis due to its concurrent sequencing, counting, and accurate assembly of millions of DNA reads similar with NGS method [26, 27]. Additionally, the advantage of SSP-PGD/S is its lower cost, which would allow IVF couples to have more samples tested for choosing the most competent embryos to transfer, comparing with the same cost of array-CGH. The features above indicate its clinical usefulness for the future.

## Conclusions

This study aimed to evaluate the applicability and efficiency of SSP-PGD/S method. The two commercial WGA kits demonstrated high compatibility with our SSP method. In the preliminary preclinical study, SSP-PGD/S and array-CGH demonstrated a 100% consistency for negative and positive diagnosis in comprehensive aneuploidy detection. In addition, SSP-PGD/S method also have potential advantages in diagnosing microdeletions, microduplication, and chimerism. Finally, due to its high efficiency and low price, our results support the SSP as an effective substitute for CGH.

## Additional file

Additional file 1: **Table S1.** Information on the artificial single-cell-like DNA samples. **Table S2.** Characteristics of the IVF-PGD couples involved in the study and their clinical outcomes. **Table S3.** Non-concordant positive results of blastocysts detected by NGS and array-CGH screening. **Table S4.** Abnormal karyotype in parents. Table S5 Karyotype of embryos. **Figure S1.** Representation samples of NGS results from chromosomally unbalanced embryos and patients with abnormal chromosome. (DOCX 881 kb)

## Abbreviations

AFA: Adaptive Focused Acoustics
array-CGH: Array comparative genomic hybridization
CNVs: Copy number variations
CV: Coefficients of variance
FISH: Fluorescence in situ hybridization
IVF: In vitro fertilization
NGS: Next-generation sequencing
PGS/D: Preimplantation genetic screening/diagnosis
SSP: Semiconductor sequencing platform
WGA: Whole-genome amplification
