# Supplementary material for: Preimplantation genetic diagnosis and screening (PGD/S) using a semiconductor sequencing platform
Source: Hum Genomics. 2019 Jan 3;13:1. doi: 10.1186/s40246-018-0187-x (PMC6318931; doi:10.1186/s40246-018-0187-x)
Supplement: Supplementary file 1 — Table S1. Information on the artificial single-cell-like DNA samples. Table S2. Characteristics of the IVF-PGD couples involved in the study and their clinical outcomes. Table S3. Non-concordant positive results of blastocysts detected by NGS and array-CGH screening. Table S4. Abnormal karyotype in parents. Table S5 Karyotype of embryos. Figure S1. Representation samples of NGS results from chromosomally unbalanced embryos and patients with abnormal chromosome. (DOCX 881 kb) [file 40246_2018_187_MOESM1_ESM.docx]

**Table S1** Information on the artificial single-cell-like DNA samples

| **Sample No.** | **Karyotype** | **Stock** |
| --- | --- | --- |
| 1 | 47,XX,+14 | DNA |
| 2 | 47,XX,+7 | DNA |
| 3 | 46,XY,dup3 (2.18Mb) | DNA |
| 4 | 46,XN,del3 (2.88Mb) | DNA |
| 5 | 46,XY,dup18(3.02Mb) | DNA |
| 6 | 46,XX,dup20 (3.14Mb) | DNA |
| 7 | 46,XX,dup5(121.24Mb),del18(8.58Mb) | DNA |
| 8 | 47,XY,+18[3]/ 47,XY,+15[6] | DNA |
| 9 | 47,XY,+14[3]/ 47,XY,+13[6] | DNA |
| 10 | 46,XX,dup11(15.3Mb),del11(3.34Mb)[3]/ 47,XY,+14[6] | DNA |
| 11 | 47,XX,+13[3]/ 46,XX,dup4(152.1Mb),del22(4.86Mb)[6] | DNA |
| 12 | 47,XY,+15[3]/ 46,XY,dup11(18.2Mb),dup22(4.02Mb)[6] | DNA |
| 13 | 46,XX,dupX(20.5Mb),delX(62Mb)[3]/ 46,XY,+18[6] | DNA |
| 14 | 46,XX,dup5(121.24Mb),del18(8.58Mb)[3]/ 46,XX,del11(13.46Mb)[6] | DNA |
| 15 | 46,XX,del18(6.32Mb)[3]/ 46, XX,dup5(121.24Mb),del18(8.58Mb)[6] | DNA |
| 16 | 46,XY,del3(8.5Mb),dup12(27.6Mb)[3]/ 46,XY,dup2(54.5Mb)[6] | DNA |
| 17 | 46,XY,dup4(30.58Mb),del14(19.68Mb)[3]/ 46,XY,del3(8.5Mb),dup12(27.6Mb)[6] | DNA |
| 18 | 46,XX,del11(13.46Mb)[3]/ 46,XX,del18(6.32Mb)[6] | DNA |
| 19 | 46,XY,dup2(54.5Mb)[3]/ 46,XY,dup4(30.58Mb),del14(19.68Mb)[6] | DNA |
| 20 | Normal male single B lymphocytes | Single cell |

**Table S2** Characteristics of the IVF-PGD couples involved in the study and their clinical outcomes.

| **Parameters** | | **Results** |
| --- | --- | --- |
| No. of couples treated | | 157 |
| Classifications^1^ | | Translocation (73), Rob (39), XY abnormality (13), inversion (13), PGS(19); |
| Mean female age ± SD | | 30.27±4.08 |
| No. of blastomeres diagnosed | | 543 |
| No. of samples biopsied | No. of cycles: 1 | 150 |
|  | No. of cycles: 2 | 11 |
|  | No. of cycles: 3 | 1 |

**Table S3** Non-concordant positive results of blastocysts detected by NGS and array-CGH screening.

| **Samples** | **NGS** | **Array-CGH** |
| --- | --- | --- |
| 6-1 | 47,XXY,dup(2)(p11.2-q24.2) | 48,XXY,+21,dup(2)(p11.2-q24.2) |
| 8-1 | 47,XXY, | 52,XXY,+1,+8,+9,+10,+16,+18,+20,-15,-22 |
| 8-2 | 45,XY,-22 | 47,XY,+4,+8,-22 |
| 8-3 | 47,XXY,+6,-16 | 57,XXY,+1,+2,+6,+7,+8,+9,+11,+14,+17,+18,+20,-22 |
| 12-2 | 45,XX,-14,dup(13)(q31.3-qter) | 46,XX,-14,+16,dup(13)(q31.3-qter) |
| 21-5 | 45,XY,-12,dup(1)(pter-p34.3),del(5)(pter-q14.1) | 46,XY,+1,-12,del(5)(pter-q14.1) |
| 44-2 | 47,XY,+20,dup(6)(q22.1-qter),dup(12)(q13.11-qter),dup(15)(q11.2-qter) | 48,XY,+15,+20,dup(6)(q22.1-qter),dup(12)(q13.11-qter) |
| 56-4 | 42,XX,-1,-3,-13,-20 | 43,XX,-1,-3,-13,-20,+21 |
| 72-2 | 45,XY,+14,-13,dup(15)(q14-q25.2) | 46,XY,+9,+14,-13,+15 |
| 74-3 | 45,XY,+9,-1,-4,del(15)(q11.2-qter) | 46,XY,+9,+22,-1,-4,del(15)(q11.2-qter) |
| 80-5 | 46,XX,dup(1)(pter-p35.3),del(9)(q21.11-qter) | 45,XX,-9,dup(1)(pter-p35.3) |
| 110-5 | 46,XY,dup(1)(pter-p31.1),dup(8)(q23.3-q24.21) | 47,XY,+22,dup(1)(pter-p31.1),dup(8)(q23.3-q24.21) |
| 118-3 | 49,XXY,+2,+16,dup(6)(q22.31-qter),dup(11)(q14.1-qter),dup(3)(q26.33-qter) | 48,XXY,+2,+16,-19,dup(6)(q22.31-qter),dup(11)(q14.1-qter),dup(3)(q26.33-qter) |
| 139-2 | 46,XY,dup(8)(pter-p12) | 45,XY,-12,dup(8)(pter-p12) |

**Table S4** Abnormal karyotype in parents

| **Parents ID** | **Abnormal karyotype in parents** |
| --- | --- |
| 1 | 46,XY,inv(1)(p13;q21) |
| 2 | 46,XX,t(4;18) |
| 3 | 45,XX,der(13;14)(q10;q10) |
| 4 | 45.XY,der(13;14)(q10;q10) |
| 5 | 47,XYY |
| 6 | 45,XX,rob(13;14) |
| 7 | 46,XY,t(6;21)(p21.3;q22) |
| 8 | NA |
| 9 | 46,XX,t(11;20)(p15;p11) |
| 10 | NA |
| 11 | 45,XY,rob(14;15) |
| 12 | 45,XX,rob(13;14) |
| 13 | NA |
| 14 | 47,X,inv(Y)*2 |
| 15 | 45,XY,rob(13;14) |
| 16 | 46,XX,inv(10) |
| 17 | 46,XX,t(3;15)(q13.1;q26.3) |
| 18 | 46,XX,t(1;9)(p31;p24) |
| 19 | 45,XY,t(15;14)(p11;q11) |
| 20 | 46,XY,t(3;9)(p14;q32) |
| 21 | 46,XX,t(12;10)(q15;q24) |
| 22 | 46,XX,t(1;3)(p21;q21) |
| 23 | 46,XY,t(4;16)(q21;q22) |
| 24 | 45,XY,rob(13;14) |
| 25 | 46,XX,t(2;7)(p10;q10) |
| 26 | 45,XY,der(13;22) |
| 27 | 47,XXX |
| 28 | 46,XY,t(5;14)(q22;q32) |
| 29 | 45,XY,t(14;21) |
| 30 | 45,XX,der(13;14) |
| 31 | 46,XY,t(11;14) |
| 32 | 45,XX,der(14;15) |
| 33 | 47.XYY |
| 34 | 45,XY,rob(13;14) |
| 35 | 46,XY,t(3;4)(q24;q28) |
| 36 | 46,XY,t(4;10) |
| 37 | 45,XY,t(13;14) |
| 38 | 45,X,(4)/46,XX(46) |
| 39 | NA |
| 40 | 46,XY,inv(3) |
| 41 | 46,XX,t(3;7)(p12;q31) |
| 42 | 46,XX,t(3;6) |
| 43 | NA |
| 44 | 46,XY,t(10;19)(q25;q13.3) |
| 45 | 45,XY,t(15;22) |
| 46 | 46,XY,t(2;7) |
| 47 | 45,XX,t(21;22) |
| 48 | NA |
| 49 | 46,XY,t(1;11)(q22;p14) |
| 50 | 46,XX,t(6;8) |
| 51 | 46,XX,t(1;4)(p32;p15.3) |
| 52 | 46,XY,t(4;22)(q24;q13) |
| 53 | 45,XY,rob(13;14)(q10;q10) |
| 54 | 46,XX,t(13;18)(q22;q21.1) |
| 55 | 46,XY,t(13;14) |
| 56 | 46,XY,inv(4) |
| 57 | 46,XY,t(5;9)(p15.3;p12) |
| 58 | NA |
| 59 | 46,XY,t(6;13)(q21;q33) |
| 60 | 46,XY,t(1;2)(p31;p23) |
| 61 | 45,XX,rob(13;14) |
| 62 | 46,XX,t(9;11) |
| 63 | NA |
| 64 | 45,XY,der(14;21) |
| 65 | 46,XY,inv(6)(p25;p21.3) |
| 66 | 46,XY,inv(7) |
| 67 | 46,XX,t(8;17) |
| 68 | 46,XY,t(8;22) |
| 69 | 46,XX,t(5;18) |
| 70 | 45,XY,t(14;22) |
| 71 | 45,XX,der(13;14)(q10;q10) |
| 72 | 47,XYY |
| 73 | 45,XX,der(13;14) |
| 74 | 46,XY,t(1;9)(p31;p24) |
| 75 | 45,X,(10%)/46,XX(90%) |
| 76 | 45,XY,t(13;14) |
| 77 | 46,XX,t(1;4) |
| 78 | 46,XY,t(3;6) |
| 79 | 46,XY,t(9;17)(q22;p11.2) |
| 80 | 46,XY,t(1;2) |
| 81 | 45,XY,t(1;8) |
| 82 | 46,XY,t(19;22) |
| 83 | 46,XX,t(10;17)(q26;q21) |
| 84 | 45,XY,t(13;14) |
| 85 | NA |
| 86 | 46,XY,t(8;10) |
| 87 | 46,XX,t(2;3)(p10;q10) |
| 88 | 45,XY,t(14,21) |
| 89 | 46,XY,(29)/46,XY,chrg(16)(q22)(12)/46,XY,chrb(16)(q22)(9) |
| 90 | 46,XX/46,XX,t(1;5)(p11;p13) |
| 91 | 46,XX,t(1;10)(q32;p11.2) |
| 92 | 46,XX,t(9;20)(p10;q10) |
| 93 | 46,XX,t(3;18) |
| 94 | 45,XY,rob(13;14) |
| 95 | 46,XY,t(7;10)(q36;p11) |
| 96 | 46,XY,t(10;13) |
| 97 | 46,XY,t(6;17) |
| 98 | 46,XY,t(9;12) |
| 99 | NA |
| 100 | 46,XX,t(2;22)(q13;q11.2) |
| 101 | 46,XX,t(5;18) |
| 102 | 46,XY,inv(1) |
| 103 | 46,XX,t(3;7)(p14;q31) |
| 104 | 45,XX,der(13;14) |
| 105 | AZF |
| 106 | 46,XY,inv(5) |
| 107 | 46,XY,t(6;13)(q22;q14) |
| 108 | 46,XY,t(10;20)(q14;q13) |
| 109 | 46,XY,t(2;20)(p10;q10) |
| 110 | NA |
| 111 | 46,XY,t(7;14)(p13;q24) |
| 112 | 45,XY,t(15;22) |
| 113 | 45,XX,t(13;14) |
| 114 | NA |
| 115 | 46,XY,t(2;3) |
| 116 | 45,XY,t(13;14) |
| 117 | 46,XX,t(7;16)(q32;p13.2) |
| 118 | 46,XX,t(1;21)(q21;q22) |
| 119 | 46,XX,t(6;8) |
| 120 | NA |
| 121 | 46,XX,der(13;14)(q10;q10) |
| 122 | 46,XX,t(6;11) |
| 123 | 46,XY,inv(1) |
| 124 | 46,XY[48]/45,X[2] |
| 125 | 46,XY,t(5;7)(p12.3;q21) |
| 126 | 46,XY,t(4;2) |
| 127 | 46,XX,t(4;6)(q31.3;q25) |
| 128 | 46,XY,t(3;5) |
| 129 | 47,XYY |
| 130 | NA |
| 131 | 45,XY,t(13;14) |
| 132 | 46,XX,t(5;6)(p15.1;q21) |
| 133 | NA |
| 134 | 46,XY,t(5;7) |
| 135 | 46,XY,t(9;6)(q13;q33) |
| 136 | 45,XY,der(13;14)(q10;q10) |
| 137 | 46,X,i(X)(q10) |
| 138 | 47,XYY |
| 139 | NA |
| 140 | 46,XX,inv(1)(q34.3q21) |
| 141 | NA |
| 142 | 45,X[4]/46,XX[46] |
| 143 | 45,XY,rob(13;14) |
| 144 | 45,XX,rob(13;14) |
| 145 | 45,XX,der(13;15)(q10;q10) |
| 146 | 46,XY,t(4;10) |
| 147 | 46,XX,t(1;12)(p32;q24.3) |
| 148 | 46,XY,t(1;17)(q23;p13) |
| 149 | 46,XY,t(1;16) |
| 150 | 45,XY,inv(9) |
| 151 | 46,XY,t(14;17) |
| 152 | 45,XY,der(13;14)(q10;q10) |
| 153 | 45,X,(16.7%)/46,XX(83.3%) |
| 154 | 46,XY,t(3;7)(p21;p15) |
| 155 | NA |
| 156 | 46,XX,invins(7)(p21;q36) |
| 157 | 45,XY,der(15;22) |

**Table S5** Karyotype of embryos

| **Embryo ID** | **Normal karotype** | **Abnormal karotype** | | | | | |
| --- | --- | --- | --- | --- | --- | --- | --- |
|  |  | **Trisomy** | **Mosomy** | **Duplication** | **Deletion** | **CNVs** | **CNV length (Mb)** |
| 1-1 | 46,XX |  |  |  |  |  |  |
| 1-2 |  |  | D1 |  |  |  |  |
| 1-3 |  | T12 |  | dup9 | del2 del5 del13 | dup9 del2 del5 del13 | 28 110 82 58 |
| 1-4 |  | XXY |  |  |  |  |  |
| 2-1 |  | T15 T22 XXY |  | dup18 | del4 | dup18 del4 | 15 39 |
| 2-2 |  | T22 | D4 D5 D8 D9 D17 D19 |  |  |  |  |
| 2-3 |  |  |  | dup4 | del18 | dup4 del18 | 39 15 |
| 2-4 |  |  |  | dup18 | del4 | dup18 del4 | 14 39 |
| 2-5 |  |  | D16 |  | del4 | del4 | 30 |
| 2-6 |  |  | D16 | dup18 | del4 | dup18 del4 | 14 39 |
| 3-1 |  | T14 |  |  |  |  |  |
| 3-2 |  |  | D12 |  |  |  |  |
| 3-3 |  |  | D1 |  |  |  |  |
| 3-4 |  | T10 |  |  |  |  |  |
| 4-1 | 46,XY |  |  |  |  |  |  |
| 4-2 |  | T3 T5 T6 T10 T11 T14 T17 T20 T22 XXY |  |  |  |  |  |
| 4-3 | 46,XY |  |  |  |  |  |  |
| 5-1 |  | XXY | D16 | dup8 | del11 | dup8 del11 | 17 22 |
| 5-2 |  |  | D16 |  |  |  |  |
| 5-3 | 46,XX |  |  |  |  |  |  |
| 6-2 | 46,XX |  |  |  |  |  |  |
| 6-3 |  |  | D22 | dup20 | del10 del17 del22 | dup20 del10 del17 del22 | 19 57 21 34 |
| 6-4 |  |  |  |  | del16 | del16 | 42 |
| 6-5 | 46,XX |  |  |  |  |  |  |
| 6-6 | 46,XY |  |  |  |  |  |  |
| 6-7 |  | T14 | D22 |  |  |  |  |
| 6-8 |  |  | D21 |  | del3 | del3 | 86 |
| 7-1 |  |  |  |  | del6 | del6 | 128 |
| 7-2 |  |  |  | dup6 | del6 del21 | dup6 del6 del21 | 49 122 34 |
| 7-3 |  |  | D11 | dup6 | del21 | dup6 del21 | 48 22 |
| 7-4 |  |  |  | dup6 | del6 del21 | dup6 del6 del21 | 49 122 22 |
| 7-5 |  |  |  | dup6 |  | dup6 | 122 |
| 7-6 |  |  |  | dup6 |  | dup6 | 18 |
| 7-7 |  | XXY | D12 D22 | dup6 |  | dup6 | 123 |
| 7-8 |  | T7 T16 XXY | D2 D8 |  | del6 | del6 | 50 |
| 8-4 |  |  |  |  | del13 | del13 | 96 |
| 8-5 | 46,XY |  |  |  |  |  |  |
| 9-1 | 46,XY |  |  |  |  |  |  |
| 9-2 |  |  |  | dup20 |  | dup20 | 16 |
| 9-3 |  | XXY |  |  |  |  |  |
| 9-4 |  | T1 T2 T4 T13 T14 T18 T20 T21 | D10 |  |  |  |  |
| 9-5 |  |  |  | dup1 | del20 | dup1 del20 | 102 16 |
| 9-6 | 46,XY |  |  |  |  |  |  |
| 9-7 |  |  |  |  | del1 del20 | del1 del20 | 87 15 |
| 10-1 | 46,XY |  |  |  |  |  |  |
| 10-2 | 46,XY |  |  |  |  |  |  |
| 10-3 | 46,XX |  |  |  |  |  |  |
| 11-1 | 46,XX |  |  |  |  |  |  |
| 11-2 | 46,XX |  |  |  |  |  |  |
| 11-3 | 46,XX |  |  |  |  |  |  |
| 11-4 |  | T1 T5 T6 T11 T13 T14 T16 T18 T20 T21 T22 XXYY | D7 D10 |  |  |  |  |
| 12-1 |  |  | D14 |  |  |  |  |
| 12-3 |  | T14 XXY | D13 | dup1 |  | dup1 | 10 |
| 12-4 |  | T8 | D13 |  |  |  |  |
| 12-5 |  | T2 T9 | D1 | dup10 dup12 dup6 |  | dup10 dup12 dup6 | 92 38 24 |
| 13-1 |  | T1 | D2 | dup6 | del9 del16 | dup6 del9 del16 | 28 56 41 |
| 14-1 |  | T1 T6 T10 T16 T20 T21 T22 T19 T3 | D2 D17 |  |  |  |  |
| 14-2 |  | T6 | D4 D7 D11 D17 D18 XO |  |  |  |  |
| 14-3 |  | T1 T2 T3 T4 T6 T18 XXYY | D9 D11 D15 D21 |  |  |  |  |
| 15-1 |  | T21 |  |  |  |  |  |
| 15-2 |  |  | D16 |  |  |  |  |
| 15-3 | 46,XY |  |  |  |  |  |  |
| 15-4 |  |  |  | dup5 | del17 | dup5 del17 | 16 26 |
| 15-5 |  |  | D13 D21 |  |  |  |  |
| 15-6 |  |  | D13 D16 D20 D21 |  | del3 | del3 | 129 |
| 15-7 | 46,XX |  |  |  |  |  |  |
| 16-1 | 46,XX |  |  |  |  |  |  |
| 16-2 | 46,XY |  |  |  |  |  |  |
| 16-3 | 46,XX |  |  |  |  |  |  |
| 16-4 |  |  |  |  | del2 del12 | del2 del12 | 142 41 |
| 16-5 |  |  | D20 | dup2 |  | dup2 | 143 |
| 16-6 |  |  |  | dup9 |  | dup9 | 70 |
| 17-1 |  |  |  | dup3 |  | dup3 | 91 |
| 17-2 |  | T15 | D9 D21 |  |  |  |  |
| 18-1 |  |  |  | dup1 |  | dup1 | 68 |
| 18-2 |  |  | D9 D10 D19 D22 | dup1 |  | dup1 | 68 |
| 19-1 |  |  | D14 |  |  |  |  |
| 19-2 |  | T14 T10 | D15 D22 |  |  |  |  |
| 19-3 |  |  | D5 |  |  |  |  |
| 19-4 |  |  |  | dup1 |  | dup1 | 25 |
| 20-1 |  |  |  |  | del3 del9 | del3 del9 | 145 25 |
| 20-2 |  |  | D1 D13 | dup20 | del5 del11 del20 | dup20 del5 del11 del20 | 26 100 54 33 |
| 21-1 |  |  | D4 | dup9 dup12 | del12 | dup9 dup12 del12 | 43 85 48 |
| 21-2 |  | T15 | D12 |  |  |  |  |
| 21-3 |  |  |  | dup12 | del3 | dup12 del3 | 44 53 |
| 21-4 |  | T4 T6 |  | dup15 |  | dup15 | 66 |
| 21-6 |  |  |  |  | del9 | del9 | 17 |
| 21-7 |  | T15 |  | dup12 |  | dup12 | 45 |
| 22-1 |  |  |  | dup1 | del3 | dup1 del3 | 95 87 |
| 22-2 |  |  |  | dup1 | del3 | dup1 del3 | 114 87 |
| 22-3 | 46,XX |  |  |  |  |  |  |
| 23-1 | 46,XY |  |  |  |  |  |  |
| 23-2 |  |  |  | dup4 | del16 | dup4 del16 | 106 15 |
| 24-1 | 46,XY |  |  |  |  |  |  |
| 24-2 |  |  | D13 D14 |  |  | D13 D14 | 96 87 |
| 24-3 |  |  | XO |  |  |  |  |
| 24-4 |  |  |  | dup13 | del21 | dup13 del21 | 96 34 |
| 24-5 |  | T13 |  |  |  |  |  |
| 24-6 |  | T14 |  |  |  |  |  |
| 25-1 |  | T16 T22 |  | dup2 | del7 | dup2 del7 | 98 58 |
| 26-1 | 46,XY |  |  |  |  |  |  |
| 26-2 |  | XXY |  |  |  |  |  |
| 26-3 | 46,XX |  |  |  |  |  |  |
| 26-4 |  |  |  |  | del4 | del4 | 39 |
| 26-5 |  |  | D8 |  |  |  |  |
| 27-1 | 46,XY |  |  |  |  |  |  |
| 28-1 |  |  |  | dup14 | del5 | dup14 del5 | 11 58 |
| 28-2 | 46,XY |  |  |  |  |  |  |
| 28-3 |  |  |  | dup5 | del14 | dup5 del14 | 58 10 |
| 28-4 |  | T17 |  |  | del5 del6 | del5 del6 | 57 17 |
| 28-5 |  |  |  | dup5 dup14 | del5 del14 | dup5 dup14 del5 del14 | 122 10 58 77 |
| 28-6 |  |  |  | dup5 | del14 | dup5 del14 | 58 10 |
| 29-1 |  | T3 T6 T7 T14 T15 T22 | D4 D5 D8 D16 D17 DY DX |  |  |  |  |
| 29-2 |  | T1 T2 T7 T9 T11 T12 T13 T19 T22 XXY T16 | D3 D8 D15 D18 D20 D18 |  |  |  |  |
| 29-3 |  |  |  | dup17 |  | dup17 | 52 |
| 29-4 |  | XXY | D22 |  |  |  |  |
| 29-5 |  | T20 |  |  |  |  |  |
| 29-6 |  | XXY |  |  |  |  |  |
| 29-7 |  | T17 T19 | D22 |  |  |  |  |
| 29-8 |  | T1 T3 T7 T13 T19 T20 T16 | D2 |  |  |  |  |
| 29-9 | 46,XY |  |  |  |  |  |  |
| 29-10 |  | T2 T3 T4 T5 T6 T8 T10 T11 T17 T19 XXX | D12 |  |  |  |  |
| 29-11 |  |  | D12 |  |  |  |  |
| 29-12 | 46,XX |  |  |  |  |  |  |
| 30-1 |  |  |  | dup9 dup13 |  | dup9 dup13 | 114 36 |
| 30-2 |  | T14 |  |  |  |  |  |
| 31-1 |  | T22 | D14 | dup11 |  | dup11 | 49 |
| 31-2 |  | T4 T6 T7 T9 T12 T13 T14 T17 T18 T19 T22 |  |  | del11 | del11 | 49 |
| 32-1 |  | XXX |  | dup2 dup3 dup16 | del1 del8 del9 del17 del6 | dup2 dup3 dup16 del1 del8 del9 del17 del6 | 56 87 41 103 39 63 22 54 |
| 32-2 |  |  | D9 |  | del17 | del17 | 12 |
| 32-3 |  |  |  | dup2 |  | dup2 | 95 |
| 32-4 |  | T14 XXY | D19 |  | del5 | del5 | 15 |
| 32-5 |  | T16 | D14 D15 | dup2 | del7 | dup2 del7 | 73 48 |
| 33-1 |  |  |  |  | del2 | del2 | 59 |
| 34-1 |  | T19 |  |  |  |  |  |
| 34-2 |  |  | D5 D7 D11 D12 D20 XO |  | del8 | del8 | 59 |
| 35-1 |  |  |  | dup4 dup9 | del3 | dup4 dup9 del3 | 131 70 151 |
| 35-2 |  |  |  |  | del6 | del6 | 97 |
| 36-1 |  |  | D16 | dup2 dup4 dup10 dup11 | del1 del10 | dup2 dup4 dup10 dup11 del1 del10 | 142 77 49 25 103 86 |
| 36-2 |  |  | D22 | dup4 | del10 | dup4 del10 | 114 49 |
| 36-3 |  | T1 T3 T5 T6 T9 T11 T13 T15 T18 T22 XXX |  |  |  |  |  |
| 37-1 | 46,XX |  |  |  |  |  |  |
| 37-2 | 46,XX |  |  |  |  |  |  |
| 38-1 | 46,XX |  |  |  |  |  |  |
| 38-2 |  |  | D20 |  |  |  |  |
| 38-3 |  | T14 | D5 D8 D11 D15 D18 D19 D21 XO D12 |  |  |  |  |
| 38-4 | 46,XX |  |  |  |  |  |  |
| 38-5 |  | T5 T15 T22 |  | dup13 |  | dup13 | 22 |
| 39-1 | 46,XX |  |  |  |  |  |  |
| 39-2 |  | T3 T5 T7 T8 T10 T12 T18 | D11 D20 D17 |  |  |  |  |
| 39-3 | 46,XX |  |  |  |  |  |  |
| 39-4 |  |  | D15 D17 D22 |  | del6 | del6 | 48 |
| 40-1 | 46,XY |  |  |  |  |  |  |
| 40-2 |  |  | D22 |  | del2 | del2 | 125 |
| 41-1 |  |  | D13 | dup3 | del7 | dup3 del7 | 45 57 |
| 41-2 |  |  | D22 | dup7 | del3 del14 | dup7 del3 del14 | 60 45 81 |
| 42-1 |  | XXY | D11 | dup1 |  | dup1 | 12 |
| 42-2 |  |  | D3 |  | del6 | del6 | 29 |
| 42-3 |  | T1 T2 T6 T8 T11 T17 XXX | D5 D15 |  | del3 del18 | del3 del18 | 37 32 |
| 42-4 |  | T1 T7 T9 T13 T16 T17 T19 T22 | D4 | dup3 | del6 | dup3 del6 | 33 29 |
| 43-1 |  |  | D8 D17 D20 D6 |  | del12 | del12 | 27 |
| 43-2 |  | XXY |  |  |  |  |  |
| 43-3 |  |  | D1 D3 D6 |  |  |  |  |
| 44-1 |  | XXY T5 |  | dup19 dup20 | del1 del7 del9 del10 | dup19 dup20 del1 del7 del9 del10 | 22 33 129 47 70 46 |
| 44-3 |  |  |  | dup19 | del10 | dup19 del10 | 8 19 |
| 45-1 |  |  | D15 |  |  |  |  |
| 45-2 |  | XXY |  |  |  |  |  |
| 45-3 |  |  | D4 |  |  |  |  |
| 46-1 |  |  |  |  | del7 del2 | del7 del2 | 65,216 |
| 46-2 |  |  | D2 | dup7 | del16 | dup7 del16 | 93 19 |
| 47-1 |  |  | D21 |  |  |  |  |
| 47-2 |  | T12 | D22 |  |  |  |  |
| 47-3 |  |  | D16 |  |  |  |  |
| 47-4 |  | T21 | D8 | dup12 |  | dup12 | 19 |
| 48-1 | 46,XY |  |  |  |  |  |  |
| 49-1 |  | T5 T7 T10 XXX T9 | D2 D3 D14 D18 D22 D12 | dup1 dup16 dup21 dup6 dup8 | del1 del11 del17 del15 | dup1 dup16 dup21 dup6 dup8 del1 del11 del17 del15 | 45 35 18 54 25 103 56 56 79 |
| 49-2 |  | T22 | D1 D5 D6 D7 D8 D11 D13 D14 D16 D17 XO |  |  |  |  |
| 49-3 |  | T1 | D14 |  |  |  |  |
| 49-4 |  |  | D1 D4 D20 D11 | dup16 dup17 | del8 del15 | dup16 dup17 del8 del15 | 23 32 59 15 |
| 50-1 |  |  |  | dup6 |  | dup6 | 34 |
| 50-2 |  |  | D5 D15 D19 | dup6 |  | dup6 | 29 |
| 50-3 |  |  |  | dup6 |  | dup6 | 29 |
| 50-4 |  |  | D2 D7 D11 D14 D15 D16 D17 D20 D22 |  | del6 | del6 | 29 |
| 51-1 |  | T4 | D22 |  |  |  |  |
| 51-2 |  | XXY |  |  |  |  |  |
| 51-3 |  | T4 |  |  |  |  |  |
| 52-1 |  | T18 |  |  | del14 del22 | del14 del22 | 33 34 |
| 52-2 |  |  | D1 D3 D4 D7 D9 D10 D17 D18 D19 XO | dup22 | del14 | dup22 del14 | 7 33 |
| 52-3 |  | T6 T8 T16 | D2 D3 D4 D5 D7 D10 D22 |  |  |  |  |
| 52-4 |  | XXY |  |  |  |  |  |
| 52-5 |  | T2 T3 T15 T20 | D22 |  |  |  |  |
| 52-6 |  |  |  |  | del14 | del14 | 33 |
| 52-7 | 46,XX |  |  |  |  |  |  |
| 53-1 | 46,XX |  |  |  |  |  |  |
| 53-2 |  |  | D6 D8 D15 D20 |  |  |  |  |
| 53-3 |  |  | D14 D1 |  |  |  |  |
| 53-4 |  |  |  |  | del22 | del22 | 34 |
| 53-5 |  | T14 XXY | D3 D7 D8 D11 D16 D19 |  |  |  |  |
| 53-6 |  | T17 YY | D2 D3 D19 |  |  |  |  |
| 54-1 |  | T13 | D2 |  |  |  |  |
| 54-2 |  |  |  |  | del13 del18 | del13 del18 | 42 47 |
| 54-3 |  |  |  | dup8 |  | dup8 | 57 |
| 54-4 |  | T1 T2 T4 T9 T11 T12 T14 T16 T17 T19 XXXY | D21 |  |  |  |  |
| 54-5 |  |  |  | dup2 | del1 | dup2 del1 | 14 72 |
| 55-1 | 46,XX |  |  |  |  |  |  |
| 55-2 |  |  | D3 D4 D5 D10 D13 D16 D19 |  |  |  |  |
| 55-3 |  |  | XO D1 D10 | dup18 | del2 del7 del18 | dup18 del2 del7 del18 | 11 48 28 36 |
| 55-4 |  |  |  | dup4 dup1 | del4 | dup4 dup1 del4 | 143 47 48 |
| 56-1 |  |  |  |  | del1 del16 del17 del2 del4 del6 | del1 del16 del17 del2 del4 del6 | 34 8 7 11 14 12 |
| 56-2 |  | T6 | D8 D9 XO |  |  |  |  |
| 56-3 |  |  |  | dup4 | del4 | dup4 del4 | 132 59 |
| 57-1 |  | T5 T19 |  |  | del9 | del9 | 103 |
| 57-2 |  |  | D5 |  |  |  |  |
| 57-3 |  |  |  |  | del9 | del9 | 39 |
| 57-4 |  | T6 T19 T20 |  |  |  |  |  |
| 58-1 |  | T10 T14 XYY | D2 D6 D7 D9 D18 D21 | dup1 |  | dup1 | 28 |
| 59-1 |  |  |  | dup9 |  | dup9 | 24 |
| 60-1 |  | T8 T9 T18 XXY | D6 D21 | dup12 | del17 | dup12 del17 | 63 25 |
| 60-2 |  | T4 T5 T8 XXY | D3 D10 | dup9 |  | dup9 | 70 |
| 61-1 | 46,XX |  |  |  |  |  |  |
| 61-2 |  | T14 | D10 |  |  |  |  |
| 61-3 |  | T7 T9 T16 | D13 D18 |  | del6 | del6 | 92 |
| 61-4 |  | XXY | D14 |  |  |  |  |
| 61-5 |  |  |  | dup5 dup11 |  | dup5 dup11 | 108 16 |
| 61-6 |  |  | D13 |  |  |  |  |
| 61-7 |  |  | D14 |  |  |  |  |
| 61-8 | 46,XY |  |  |  |  |  |  |
| 61-9 | 46,XX |  |  |  |  |  |  |
| 61-10 |  | T22 | D14 D21 |  |  |  |  |
| 61-11 |  |  | D13 |  |  |  |  |
| 61-12 |  | T5 | D9 D13 |  |  |  |  |
| 62-1 |  | XXY |  |  |  |  |  |
| 62-2 |  |  |  | dup5 |  | dup5 | 9 |
| 62-3 | 46,XY |  |  |  |  |  |  |
| 63-1 |  | T21 XXY | D3 | dup1 |  | dup1 | 17 |
| 64-1 |  |  | D21 |  |  |  |  |
| 64-2 |  | T4 T7 T9 T11 T13 T15 T16 T17 T19 T20 T22 | D10 D21 |  |  |  |  |
| 64-3 |  | T21 |  | dup2 | del2 | dup2 del2 | 98 87 |
| 64-4 |  |  | D17 |  |  |  |  |
| 64-5 | 46,XY |  |  |  |  |  |  |
| 64-6 |  |  | D1 D2 D3 D4 D5 D7 D8 D14 D15 D17 D18 D21 |  |  |  |  |
| 65-1 |  |  | D14 |  | del1 | del1 | 103 |
| 65-2 |  | XXY | D16 | dup17 dup6 |  | dup17 dup6 | 32 19 |
| 65-3 |  | T6 |  |  |  |  |  |
| 65-4 |  |  | D6 D14 |  |  |  |  |
| 65-5 |  | T9 |  | dup4 dup18 dup7 dup8 | del3 del5 del11 | dup4 dup18 dup7 dup8 del3 del5 del11 | 8 40 5 18 94 96 41 |
| 65-6 | 46,XX |  |  |  |  |  |  |
| 65-7 |  |  | D19 |  |  |  |  |
| 66-1 | 46,XX |  |  |  |  |  |  |
| 66-2 | 46,XX |  |  |  |  |  |  |
| 67-1 |  | T8 |  |  |  |  |  |
| 67-2 |  | T1 T16 |  |  |  |  |  |
| 68-1 |  | T22 | D8 |  | del12 | del12 | 19 |
| 69-1 |  |  | D3 D4 D5 D6 D9 D13 D16 D18 D19 D21 |  |  |  |  |
| 69-2 |  | XXY | D6 D16 | dup20 |  | dup20 | 31 |
| 70-1 |  | T2 T1 |  |  |  |  |  |
| 71-1 |  | T14 |  |  |  |  |  |
| 71-2 |  | T13 |  |  |  |  |  |
| 72-1 |  | T1 T3 T5 T10 T16 T17 T22 | D2 D4 D6 D8 D9 D15 D21 |  |  |  |  |
| 73-1 |  |  | D4 D6 D11 D13 D20 D21 D14 |  |  |  |  |
| 73-2 |  | XXY | D13 |  |  |  |  |
| 73-3 |  | T1 T11 T14 T19 T20 XXYY | D2 D4 D8 D9 D10 |  |  |  |  |
| 73-4 |  | T8 T14 | D15 |  |  |  |  |
| 73-5 |  | T13 | D16 |  |  |  |  |
| 73-6 |  |  | D2 D14 D16 |  |  |  |  |
| 74-1 |  | XXY |  |  |  |  |  |
| 74-2 | 46,XX |  |  |  |  |  |  |
| 74-4 |  |  |  | dup19 | del1 | dup19 del1 | 21 80 |
| 74-5 |  | XXY |  |  |  |  |  |
| 74-6 |  |  |  |  | del1 | del1 | 80 |
| 75-1 |  | T5 XXY |  |  |  |  |  |
| 75-2 | 46,XY |  |  |  |  |  |  |
| 75-3 |  |  |  |  | del16 | del16 | 44 |
| 75-4 |  |  | D2 D20 |  |  |  |  |
| 75-5 | 46,XX |  |  |  |  |  |  |
| 76-1 |  |  | D4 D21 |  |  |  |  |
| 76-2 |  | T16 T17 T21 T4 T6 XXX |  |  |  |  |  |
| 77-1 |  | T4 |  | dup1 | del9 | dup1 del9 | 12 70 |
| 77-2 |  | T21 | D7 | dup1 | del4 | dup1 del4 | 12 40 |
| 77-3 |  |  | D5 D9 D15 | dup1 | del4 | dup1 del4 | 12 45 |
| 77-4 |  | T1 T3 T5 T12 T16 T17 T18 T19 XXX | D7 D8 D9 D10 D21 D22 |  |  |  |  |
| 77-5 |  | T1 T7 T10 T12 T19 | D2 D3 D4 D6 D8 D9 D14 D15 D17 D18 D20 XO |  |  |  |  |
| 77-6 |  | T4 |  |  |  |  |  |
| 77-7 |  | T4 | D1 |  |  |  |  |
| 78-1 | 46,XY |  |  |  |  |  |  |
| 78-2 |  |  |  | dup3 |  | dup3 | 86 |
| 79-1 |  |  |  | dup9 |  | dup9 | 50 |
| 79-2 |  |  |  | dup9 dup17 | del9 del17 | dup9 dup17 del9 del17 | 93 19 48 62 |
| 79-3 |  | T14 | D1 D16 |  | del7 | del7 | 33 |
| 79-4 |  |  | D16 | dup2 |  | dup2 | 27 |
| 79-5 |  | T1 T3 T8 T9 T10 T11 T13 T17 T18 T19 T22 XXY | D7 |  |  |  |  |
| 80-1 |  | T19 |  |  |  |  |  |
| 80-2 |  | T5 T6 T8 T11 T15 T16 T17 T20 T22 XXYY | D18 |  | del1 | del1 | 20 |
| 80-3 |  |  |  |  | del1 | del1 | 29 |
| 80-4 |  |  |  |  | del9 del12 | del9 del12 | 56 21 |
| 81-1 |  | T8 | D1 |  |  |  |  |
| 81-2 |  |  | D1 | dup16 |  | dup16 | 32 |
| 82-1 |  | T9 T12 T22 | D11 |  | del15 | del15 | 55 |
| 82-2 |  | T22 |  |  |  |  |  |
| 83-1 |  |  | D10 |  |  |  |  |
| 83-2 |  |  |  |  | del17 | del17 | 35 |
| 83-3 |  | T2 T3 T7 T8 T11 T14 T15 T17 T18 T22 | D4 D9 D19 |  |  |  |  |
| 83-4 |  | XXY |  |  |  |  |  |
| 84-1 | 46,XY |  |  |  |  |  |  |
| 84-2 |  |  | D19 D22 |  |  |  |  |
| 84-3 |  | T16 T22 XXY |  | dup21 dup2 |  | dup21 dup2 | 15 6 |
| 84-4 |  |  | D6 D17 |  |  |  |  |
| 85-1 |  |  | D22 |  |  |  |  |
| 85-2 |  | T2 T3 T5 T6 T10 T12 T13 T18 T19 T9 | D20 |  |  |  |  |
| 85-3 |  | T2 T8 T14 T16 T18 T19 T20 T21 |  |  |  |  |  |
| 85-4 |  | T3 T10 T12 T17 T18 | D6 D8 D14 D15 D19 D22 DX |  | del13 | del13 | 64 |
| 85-5 |  |  | D4 | dup6 dup7 |  | dup6 dup7 | 13 11 |
| 85-6 |  | XYY T17 | D20 | dup1 dup5 dup7 dup9 dup10 | del7 del16 | dup1 dup5 dup7 dup9 dup10 del7 del16 | 102 142 58 70 81 97 44 |
| 86-1 |  | T4 T8 T15 T16 T19 T20 | D6 D7 D9 D10 D12 D21 D2 |  |  |  |  |
| 86-2 | 46,XX |  |  |  |  |  |  |
| 86-3 | 46,XY |  |  |  |  |  |  |
| 86-4 |  |  | D22 | dup8 dup10 | del8 del10 | dup8 dup10 del8 del10 | 98 76 48 59 |
| 86-5 |  |  |  | dup10 | del8 | dup10 del8 | 76 48 |
| 87-1 |  |  |  | dup3 | del2 | dup3 del2 | 90,135 |
| 87-2 |  | T3 |  |  | del2 | del2 | 135 |
| 87-3 |  |  |  |  | del2 del3 | del2 del3 | 135,105 |
| 87-4 |  |  | D16 | dup2 | del3 | dup2 del3 | 105,104 |
| 87-5 |  | T4 XXY |  | dup3 | del2 | dup3 del2 | 90,141 |
| 87-6 |  | T2 |  |  | del3 | del3 | 90 |
| 87-7 |  | T9 | D2 D5 D6 D8 D10 D17 D18 D19 D20 D21 D22 |  |  |  |  |
| 87-8 | 46,XX |  |  |  |  |  |  |
| 88-1 |  |  | D22 |  |  |  |  |
| 89-1 | 46,XX |  |  |  |  |  |  |
| 89-2 |  | T6 T10 T12 T13 |  |  |  |  |  |
| 89-3 | 46,XX |  |  |  |  |  |  |
| 89-4 |  |  |  |  | del8 | del8 | 57 |
| 90-1 |  |  |  |  | del3 | del3 | 85 |
| 91-1 |  |  |  | dup10 | del1 | dup10 del1 | 16 33 |
| 91-2 |  |  |  | dup1 | del10 del1 | dup1 del10 del1 | 31 16 42 |
| 91-3 |  |  |  | dup1 | del10 | dup1 del10 | 32 16 |
| 91-4 | 46,XX |  |  |  |  |  |  |
| 91-5 |  |  |  | dup1 |  | dup1 | 12 |
| 92-1 |  |  |  | dup9 | del20 | dup9 del20 | 39 26 |
| 92-2 |  | T22 T20 | D10 |  | del9 | del9 | 81 |
| 92-3 |  |  |  | dup20 | del9 | dup20 del9 | 33 58 |
| 92-4 |  |  | D10 D16 | dup20 | del9 | dup20 del9 | 26 39 |
| 92-5 |  | T1 T2 T11 T16 T17 T20 T22 |  |  | del9 | del9 | 29 |
| 92-6 |  |  |  | dup9 dup20 | del9 del20 | dup9 dup20 del9 del20 | 70 26 39 33 |
| 92-7 |  | T12 T15 T18 T20 |  |  | del1 | del1 | 49 |
| 92-8 |  |  | D15 | dup20 dup9 | del9 | dup20 dup9 del9 | 26 70 39 |
| 93-1 |  | T4 T5 T6 T7 T11 T22 T19 XXX | D8 D20 | dup14 dup18 | del13 del1 | dup14 dup18 del13 del1 | 87 47 35 12 |
| 93-2 |  |  | D1 D12 D14 D15 D17 D21 XO |  |  |  |  |
| 93-3 |  |  |  | dup22 dup18 |  | dup22 dup18 | 34 31 |
| 93-4 |  |  |  |  | del18 | del18 | 31 |
| 93-5 |  | XXY |  |  |  |  |  |
| 93-6 |  |  |  |  | del16 | del16 | 43 |
| 93-7 |  |  | D11 |  | del14 | del14 | 24 |
| 94-1 |  |  | D7 | dup11 | del11 | dup11 del11 | 14 66 |
| 94-2 | 46,XY |  |  |  |  |  |  |
| 94-3 |  | XXY | D17 |  |  |  |  |
| 94-4 |  |  |  | dup1 |  | dup1 | 117 |
| 94-5 |  | T3 T5 T7 T9 T11 T12 T16 T17 T21 XXY | D14 D19 |  |  |  |  |
| 94-6 |  | T19 | D4 |  |  |  |  |
| 95-1 |  |  |  | dup10 | del7 | dup10 del7 | 37 6 |
| 95-2 |  |  |  | dup7 | del10 | dup7 del10 | 6 37 |
| 95-3 |  |  |  | dup7 | del10 | dup7 del10 | 7 38 |
| 96-1 |  | XXY |  | dup10 dup13 | del10 del13 | dup10 dup13 del10 del13 | 67 31 68 65 |
| 96-2 |  | T13 | D1 D5 D9 D12 D14 D19 D21 |  | del10 | del10 | 68 |
| 97-1 |  |  |  | dup6 dup17 | del2 del6 | dup6 dup17 del2 del6 | 150 10 18 21 |
| 98-1 |  |  |  | dup9 | del12 | dup9 del12 | 67 22 |
| 98-2 | 46,XY |  |  |  |  |  |  |
| 98-3 |  |  | D9 |  | del12 | del12 | 22 |
| 98-4 |  |  |  | dup9 | del12 | dup9 del12 | 63 22 |
| 99-1 |  |  |  |  | del8 | del8 | 59 |
| 99-2 | 46,XY |  |  |  |  |  |  |
| 99-3 |  |  | D16 |  |  |  |  |
| 100-1 |  | T6 T16 T8 |  | dup10 | del1 | dup10 del1 | 46 5 |
| 100-2 |  |  |  | dup2 |  | dup2 | 108 |
| 100-3 |  | T22 | D1 D14 |  | del2 | del2 | 130 |
| 101-1 |  |  | D18 | dup5 |  | dup5 | 62 |
| 101-2 |  | T5 |  |  |  |  |  |
| 102-1 |  | XXY |  |  |  |  |  |
| 102-2 | 46,XX |  |  |  |  |  |  |
| 102-3 |  | XXY | D14 D19 |  |  |  |  |
| 103-1 |  |  |  |  | del3 del7 del12 | del3 del7 del12 | 121 57 35 |
| 103-2 | 46,XX |  |  |  |  |  |  |
| 103-3 |  |  |  | dup7 | del3 | dup7 del3 | 56 77 |
| 103-4 |  |  |  | dup3 | del7 | dup3 del7 | 77 56 |
| 103-5 |  |  |  | dup7 dup9 | del3 | dup7 dup9 del3 | 57 70 77 |
| 103-6 |  |  |  | dup7 | del3 | dup7 del3 | 56 77 |
| 104-1 |  |  | D13 |  | del6 | del6 | 34 |
| 104-2 |  | T14 |  |  |  |  |  |
| 104-3 | 46,XY |  |  |  |  |  |  |
| 105-1 |  | T4 XXY |  |  |  |  |  |
| 105-2 | 46,XX |  |  |  |  |  |  |
| 106-1 |  | T10 |  | dup5 |  | dup5 | 74 |
| 106-2 |  | T3 T9 T11 T17 T7 T8 T10 | D1 D5 D22 XO |  |  |  |  |
| 106-3 |  | T3 T9 T5 T8 T18 T19 T20 | D6 D15 D16 |  |  |  |  |
| 106-4 | 46,XY |  |  |  |  |  |  |
| 106-5 |  | T19 | D8 |  |  |  |  |
| 106-6 |  |  |  |  | del1 del8 del9 del19 | del1 del8 del9 del19 | 26 32 70 38 |
| 107-1 |  | T10 | D21 D6 |  | del13 | del13 | 59 |
| 108-1 |  |  |  | dup20 | del10 | dup20 del10 | 27 38 |
| 108-2 |  | XXY |  | dup10 |  | dup10 | 39 |
| 109-1 |  | T20 |  | dup6 | del2 | dup6 del2 | 73 97 |
| 109-2 |  |  |  | dup20 | del2 | dup20 del2 | 33,157 |
| 110-1 | 46,XY |  |  |  |  |  |  |
| 110-2 |  | T3 T10 T11 |  |  |  |  |  |
| 110-3 | 46,XY |  |  |  |  |  |  |
| 110-4 | 46,XX |  |  |  |  |  |  |
| 111-1 | 46,XY |  |  |  |  |  |  |
| 112-1 |  | T2 T3 T5 T13 T14 T21 T22 XXX | D11 |  |  |  |  |
| 112-2 |  |  |  |  | del16 | del16 | 30 |
| 113-1 |  | T1 | D8 D13 | dup9 |  | dup9 | 3 |
| 114-1 |  | T7 T16 | D18 | dup1 |  | dup1 | 75 |
| 114-2 |  | T16 XXY |  |  |  |  |  |
| 114-3 |  |  |  | dup19 | del1 | dup19 del1 | 14 28 |
| 114-4 |  |  | D16 |  |  |  |  |
| 115-1 |  |  |  | dup2 | del3 | dup2 del3 | 13 39 |
| 115-2 |  | T5 |  |  |  |  |  |
| 115-3 |  |  |  | dup2 | del3 | dup2 del3 | 12 39 |
| 115-4 |  | XXY | D18 |  |  |  |  |
| 115-5 |  |  | D16 D2 | dup3 | del12 | dup3 del12 | 158 57 |
| 116-1 |  | T16 |  |  |  |  |  |
| 116-2 |  |  |  | dup10 |  | dup10 | 43 |
| 116-3 |  | XXY |  | dup2 | del8 | dup2 del8 | 92,119 |
| 116-4 |  | T14 T22 |  |  |  |  |  |
| 117-1 | 46,XY |  |  |  |  |  |  |
| 117-2 |  | T17 | D8 D10 | dup3 | del1 | dup3 del1 | 24,102 |
| 118-1 |  | XXX |  |  |  |  |  |
| 118-2 |  | T21 |  |  |  |  |  |
| 119-1 |  |  |  | dup6 | del8 del9 | dup6 del8 del9 | 36 12 103 |
| 119-2 |  | T13 T21 XXY |  |  |  |  |  |
| 119-3 |  |  | D15 D16 D18 D19 D21 | dup6 | del8 del14 | dup6 del8 del14 | 38 11 68 |
| 119-4 |  |  | D16 D21 |  | del6 | del6 | 41 |
| 120-1 |  | T22 | D21 |  |  |  |  |
| 120-2 |  | T15 T20 |  |  |  |  |  |
| 120-3 |  | T5 | D4 D12 D14 D19 D22 XO |  |  |  |  |
| 121-1 |  |  | D13 |  |  |  |  |
| 121-2 |  | T14 |  |  |  |  |  |
| 121-3 |  |  | D2 D5 D6 D9 D11 D14 D19 D20 D21 D22 |  |  |  |  |
| 121-4 |  |  |  |  | del5 | del5 | 59 |
| 122-1 |  |  | D6 | dup11 |  | dup11 | 36 |
| 122-2 |  | XXY | D20 |  |  | D20 | 63 |
| 122-3 |  | XXY |  | dup11 | del6 del11 | dup11 del6 del11 | 23 59 37 |
| 122-4 |  | XXY | D11 |  |  |  |  |
| 123-1 |  |  |  | dup1 | del1 | dup1 del1 | 16 14 |
| 123-2 |  | T15 | D20 |  | del13 del3 | del13 del3 | 27,103 |
| 123-3 |  |  | D22 |  | del1 | del1 | 16 |
| 124-1 | 46,XY |  |  |  |  |  |  |
| 124-2 | 46,XX |  |  |  |  |  |  |
| 124-3 |  |  | D6 |  |  |  |  |
| 124-4 | 46,XY |  |  |  |  |  |  |
| 125-1 |  | T1 T4 T9 T14 |  |  |  |  |  |
| 126-1 |  |  |  | dup4 | del12 | dup4 del12 | 80 91 |
| 126-2 |  |  |  |  | del12 del15 | del12 del15 | 42 79 |
| 127-1 | 46,XY |  |  |  |  |  |  |
| 127-2 | 46,XX |  |  |  |  |  |  |
| 127-3 |  |  | D6 |  | del3 del4 | del3 del4 | 104 27 |
| 128-1 |  | XXY |  |  |  |  |  |
| 128-2 |  |  | D9 |  | del3 del5 | del3 del5 | 111 43 |
| 128-3 |  | T6 T8 T11 T16 T22 | D1 D5 D7 D13 D18 DX DY |  |  |  |  |
| 129-1 |  | T19 |  |  |  |  |  |
| 129-2 |  |  |  | dup4 |  | dup4 | 54 |
| 129-3 |  |  | D17 D21 |  | delX | delX | 35 |
| 130-1 |  |  | D6 D11 |  |  |  |  |
| 130-2 |  |  | D13 D21 |  |  |  |  |
| 131-1 | 46,XY |  |  |  |  |  |  |
| 132-1 |  |  |  |  | del6 | del6 | 76 |
| 132-2 |  | T15 T5 |  | dup6 | del6 | dup6 del6 | 75 96 |
| 132-3 |  | T14 T20 T4 | D2 D6 D9 XO |  |  |  |  |
| 132-4 | 46,XX |  |  |  |  |  |  |
| 132-5 |  | XXY T18 | D3 |  |  |  |  |
| 132-6 |  |  | D9 D18 D20 |  |  |  |  |
| 133-1 |  | T9 | D10 D15 D20 |  |  |  |  |
| 133-2 |  | T15 |  |  | del10 | del10 | 28 |
| 133-3 |  |  | D9 |  |  |  |  |
| 134-1 |  |  |  | dup7 | del5 | dup7 del5 | 12 23 |
| 134-2 |  | T5 | D7 |  |  |  |  |
| 134-3 |  | T14 T22 T10 T19 T18 |  | dup3 dup9 | del2 del3 del5 del7 | dup3 dup9 del2 del3 del5 del7 | 35 70 127 32 131 64 |
| 135-1 |  |  | D21 |  | del6 | del6 | 104 |
| 135-2 |  |  |  | dup6 | del9 | dup6 del9 | 58,112 |
| 135-3 |  | XXY |  | dup6 dup10 | del9 | dup6 dup10 del9 | 81 22 113 |
| 136-1 |  | T2 T4 T8 T11 T22 |  |  |  |  |  |
| 137-1 | 46,XY |  |  |  |  |  |  |
| 137-2 | 46,XY |  |  |  |  |  |  |
| 138-1 |  | T16 T22 XXY |  | dup10 dup19 | del1 del2 | dup10 dup19 del1 del2 | 42 25 43 13 |
| 138-2 |  |  |  |  | del10 del15 | del10 del15 | 11 45 |
| 138-3 | 46,XY |  |  |  |  |  |  |
| 138-4 |  | T1 T9 T12 XXY | D7 | dup17 dup14 | del8 | dup17 dup14 del8 | 55 87 89 |
| 139-1 |  | T17 |  |  |  |  |  |
| 140-1 |  | XXX | D19 | dup7 | del7 | dup7 del7 | 112 47 |
| 140-2 |  |  |  | dup8 | del13 | dup8 del13 | 99 45 |
| 140-3 |  | T8 |  | dup1 | del1 | dup1 del1 | 90 19 |
| 140-4 |  |  |  | dup8 | del13 | dup8 del13 | 98 45 |
| 141-1 |  | T6 |  |  | del20 | del20 | 33 |
| 141-2 |  | T20 |  | dup3 dup5 | del4 del6 | dup3 dup5 del4 del6 | 45 78 128 76 |
| 141-3 |  | T11 | D1 |  | del9 | del9 | 70 |
| 141-4 |  | T7 XXY |  |  |  |  |  |
| 141-5 |  | T13 XXX | D1 D2 D3 D8 D9 D11 D18 |  |  |  |  |
| 141-6 |  | T4 T8 T10 T17 | D3 D14 | dup1 |  | dup1 | 47 |
| 142-1 | 46,XY |  |  |  |  |  |  |
| 142-2 |  | T8 T17 |  |  |  |  |  |
| 143-1 |  | T14 |  |  | del21 | del21 | 6 |
| 143-2 |  |  | D10 XO D5 D7 |  |  |  |  |
| 143-3 |  | T2 T6 T7 T8 T9 T17 T19 | DX | dup16 |  | dup16 | 69 |
| 143-4 |  | T4 | D17 | dup16 |  | dup16 | 34 |
| 144-1 |  | T14 |  | dup3 |  | dup3 | 30 |
| 144-2 |  | T14 | D2 |  | del12 | del12 | 71 |
| 144-3 |  | T3 T10 T12 T13 |  |  |  |  |  |
| 145-1 |  | T7 T19 | D15 D21 D3 |  |  |  |  |
| 145-2 |  |  | D16 | dup6 |  | dup6 | 77 |
| 145-3 |  |  |  | dup17 |  | dup17 | 49 |
| 145-4 |  | T16 XXY | D15 |  |  |  |  |
| 145-5 |  | T15 | D1 D2 D4 D7 D13 D18 D21 |  |  |  |  |
| 145-6 |  |  | D1 D2 D7 D9 D11 D18 |  |  |  |  |
| 145-7 |  | T19 | D1 D3 D10 D11 D14 D15 D17 D21 |  |  |  |  |
| 146-1 |  | T15 | D22 | dup1 | del10 | dup1 del10 | 21 81 |
| 146-2 |  |  |  | dup4 dup15 | del10 | dup4 dup15 del10 | 60 43 80 |
| 146-3 |  | XXY |  |  |  |  |  |
| 146-4 |  | T14 T16 |  | dup10 | del4 del7 | dup10 del4 del7 | 76 60 58 |
| 146-5 |  | XXY | D7 D13 D14 | dup10 dup11 | del9 | dup10 dup11 del9 | 20 56 39 |
| 146-6 |  |  |  | dup10 | del4 | dup10 del4 | 77 58 |
| 147-1 |  |  |  | dup1 | del12 | dup1 del12 | 57 31 |
| 148-1 |  | T1 | D17 |  |  |  |  |
| 148-2 | 46,XY |  |  |  |  |  |  |
| 148-3 |  |  | D20 D17 |  | del1 | del1 | 91 |
| 149-1 |  |  |  | dup7 | del1 del4 del6 | dup7 del1 del4 del6 | 45 26 73 23 |
| 149-2 |  | T3 T6 T9 T10 T18 T20 | D2 D5 D7 D8 D12 D14 D19 D1 D16 |  |  |  |  |
| 149-3 |  | XXY | D20 |  |  |  |  |
| 150-1 | 46,XX |  |  |  |  |  |  |
| 150-2 |  | T22 |  | dup7 dup2 |  | dup7 dup2 | 18 20 |
| 151-1 |  |  |  | dup17 | del14 | dup17 del14 | 34 40 |
| 151-2 |  | T14 | D17 |  |  |  |  |
| 151-3 |  |  | D14 |  |  |  |  |
| 151-4 |  |  | D1 D2 D5 D6 D8 D14 D18 XO |  | del17 | del17 | 47 |
| 152-1 |  | T5 T15 | D1 D2 D4 D6 D8 D13 D14 D16 D17 D21 DX DY |  |  |  |  |
| 152-2 |  | XXY |  |  |  |  |  |
| 152-3 |  |  |  |  | del10 del11 | del10 del11 | 41 66 |
| 153-1 |  | T14 |  |  |  |  |  |
| 153-2 | 46,XX |  |  |  |  |  |  |
| 153-3 |  | T1 T6 T10 T14 | D22 |  |  |  |  |
| 154-1 |  | T10 |  | dup3 |  | dup3 | 30 |
| 155-1 |  |  | D22 d19 |  |  |  |  |
| 155-2 | 46,XX |  |  |  |  |  |  |
| 156-1 |  |  | D4 |  | del7 | del7 | 31 |
| 157-1 |  | T7 T16 |  |  |  |  |  |
| 157-2 | 46,XX |  |  |  |  |  |  |

**
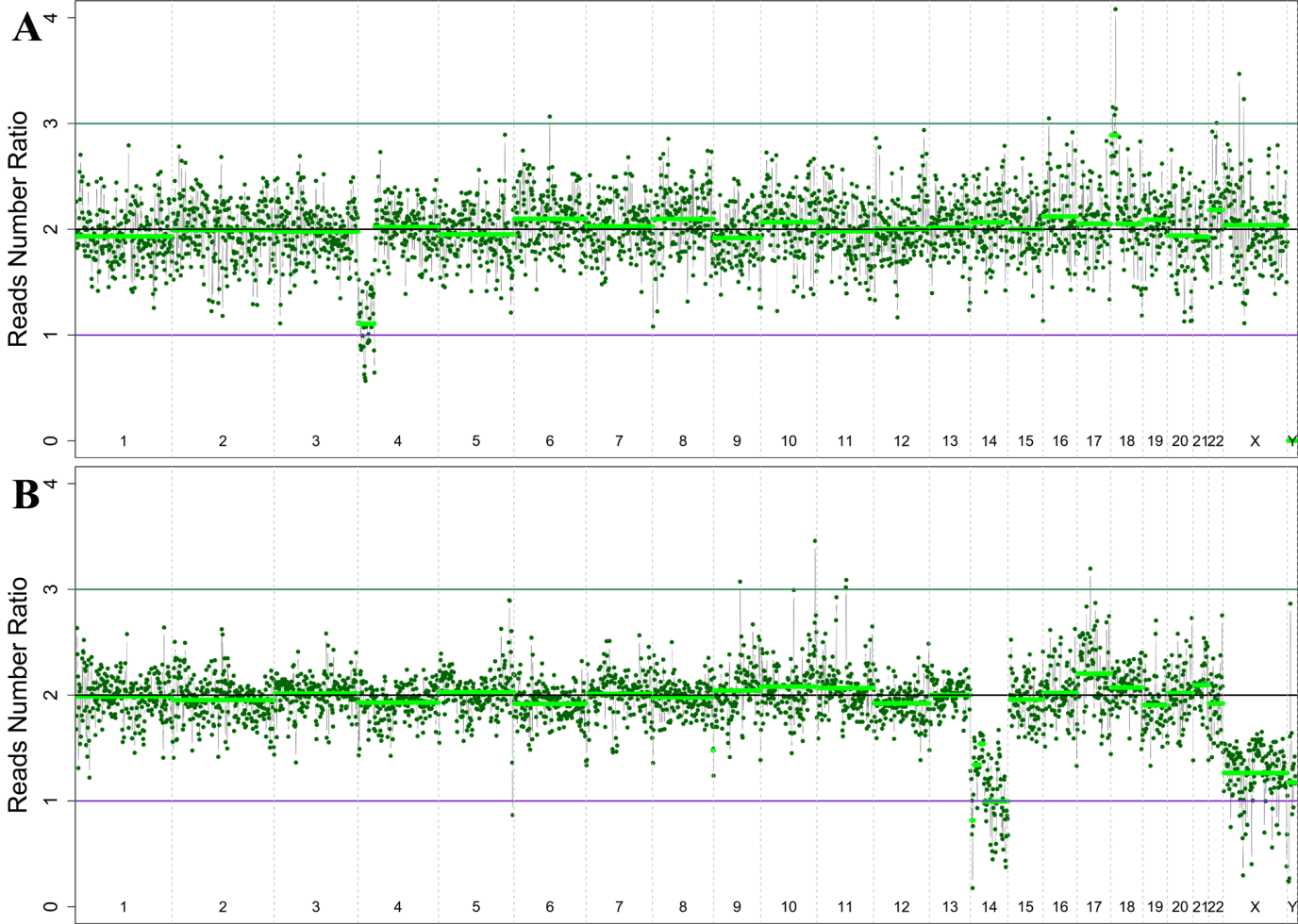
**

**Figure S1** Representation samples of NGS results from chromosomally unbalanced embryos and patients with abnormal chromosome. X-axis indicates chromosome numbers (1-22, X and Y) and y-axis indicates chromosome copy number assignments (0, 1, 2, 3, or 4). (A) Embryos biopsied derived from patients carrying a balanced translocation 46, XX, t (4;18). (B) Embryos biopsied derived from patients carrying a Robertsonian translocation 45, XX, rob (13; 14).
